# Phylogeography of the parasitic mite *Laelaps agilis* in Western Palearctic shows lineages lacking host specificity but possessing different demographic histories

**DOI:** 10.1186/s40850-022-00115-y

**Published:** 2022-03-24

**Authors:** Masoud Nazarizadeh, Jana Martinů, Milena Nováková, Michal Stanko, Jan Štefka

**Affiliations:** 1 Institute of Parasitology, Biology Centre CAS, Branišovská 31, 370 05 České Budějovice, Czech Republic; 2 Faculty of Science, University of South Bohemia, Branišovska 1760, 370 05 České Budějovice, Czech Republic; 3 Institute of Parasitology and Institute of Zoology, Slovak Academy of Sciences, Hlinkova 3, 040 01 Košice, Slovakia

**Keywords:** *Laelaps agilis*, Phylogenetic relationship, Demographic history, Host specificity

## Abstract

**Background:**

*Laelaps agilis* C.L. Koch, 1836 is one the most abundant and widespread parasitic mite species in the Western Palearctic. It is a permanent ectoparasite associated with the *Apodemus* genus, which transmits *Hepatozoon* species via the host’s blood. Phylogenetic relationships, genealogy and host specificity of the mite are uncertain in the Western Palearctic. Here, we investigated the population genetic structure of 132 individual mites across Europe from their *Apodemus* and *Clethrionomys* hosts. Phylogenetic relationships and genetic variation of the populations were analyzed using cytochrome c oxidase subunit I (COI) gene sequences.

**Results:**

We recovered three main mtDNA lineages within *L. agilis* in the Western Palearctic, which differentiated between 1.02 and 1.79 million years ago during the Pleistocene period: (i) Lineage A, including structured populations from Western Europe and the Czech Republic, (ii) Lineage B, which included only a few individuals from Greece and the Czech Republic; and (iii) Lineage C, which comprised admixed populations from Western and Eastern Europe. Contrary to their population genetic differentiation, the lineages did not show signs of specificity to different hosts. Finally, we confirmed that the sympatric congener *L. clethrionomydis* is represented by a separated monophyletic lineage.

**Conclusion:**

Differences in the depth of population structure between *L. agilis* Lineages A and C, corroborated by the neutrality tests and demographic history analyses, suggested a stable population size in the structured Lineage A and a rapid range expansion for the geographically admixed Lineage C. We hypothesized that the two lineages were associated with hosts experiencing different glaciation histories. The lack of host specificity in *L. agilis* lineages was in contrast to the co-occurring highly host-specific lineages of *Polyplax serrata* lice, sharing *Apodemus* hosts. The incongruence was attributed to the differences in mobility between the parasites, allowing mites to switch hosts more often.

**Supplementary Information:**

The online version contains supplementary material available at 10.1186/s40850-022-00115-y.

## Background

Understanding the common patterns and processes of speciation is a major goal of evolutionary biology [1, 2]. Parasites represent excellent models for studying speciation processes owing to their high mutation rates and potential in diversification and specialization [3, 4]. Population genetics and the population ecology of parasites are closely connected; for example, parasite population structures are correlated with host specificity, host mobility, infrapopulation size, reproductive mode, and life cycle complexity [4]. The relative significance of individual factors varies between different species of parasites. Thus, to understand the determining processes in the diversification of parasites, population genetic data collected across an ecologically wide range of models are needed [5–7]. 

A growing number of studies have focused on the phylogeography and population genetic relationships of rodent hosts and their associated ectoparasites showing that factors such as host switching [8–10], social structure of the hosts [11–13], and the closeness of the relationship between the host and its parasite (e.g. host specificity, [14–16]) determine the structure of parasitic populations. Using *Apodemus* mice and their *Polyplax* lice, studies of Štefka and Hypša [17] and Martinů et al. [14, 18] showed how the shared history during glaciation events in Europe affected the genetic structure of parasites. In these studies, three sympatric mitochondrial lineages were found, each with a different level of host specificity [17]. Nuclear differentiation between the lineages was confirmed [14], and within one of the lineages a cryptic hybrid zone was identified between two mitochondrial sub-clades probably originating in different glacial refugia, but sharing a single host species (*Apodemus flavicollis*) [18]. Recent genetic studies from the south African region [8, 19–21] showed how taxonomically and ecologically unrelated groups of ectoparasites, such as the *Polyplax* lice and *Laelaps* mites, may respond to parasitisation of the same host species, the *Rhabdomys* mice. Surprisingly, these studies revealed tighter co-evolutionary patterns between the facultative parasites (mites *L. giganteus* and *L. muricola*) and their hosts, rather than in the co-occurring permanent *Polyplax arvicanthis* lice.

The 12 species of mites belonging to the genus *Laelaps* (Acari: suborder Mesostigmata) are.

among the most common ectoparasites of rodents in Europe [22, 23]. Some *Laelaps* mites infect a wide variety of mammal hosts with a low degree of host specificity, whereas others tend to be more host-specific and are restricted to a limited array of mammals [20, 23, 24]. Generally, *Laelaps* mites are opportunistic feeders capable of feeding on a wide range of food items, including ectoparasites, small nest arthropods, and scabs on the skin of the hosts [25]. They may also feed directly from the hosts by creating a crater on the hosts’ skin or feed on their body fluids such as lachrymal secretions or blood [26, 27]. *Laelaps* mites are ecologically diversified; some of them occur permanently on rodent hosts while others as facultative parasites spend only part of their life cycle in the rodent fur and the rest in their nests [19, 25, 28].

*L. agilis* is an oligohostal bloodsucking and permanent mite associated mostly with the mice of the genus *Apodemus* and is widely distributed from Russia’s Far East (Khabarovsk Region) to central Asia and Europe [23]. In the Western Palearctic, this species most commonly infects two rodent species, the wood mouse *A. sylvaticus* and the yellow-necked mouse *A. flavicollis* [27, 29, 30]. It is a frequent parasite of other small ground-dwelling mammals (e.g. *Clethrionomys glareolus*, *Microtus arvalis* and *Sorex araneus*), and it is permanently present in the fur of its host [27, 31]. From an epidemiological perspective, the mite is an important vector for the transmission of the *Hepatozoon* species [29, 32]. *Borrelia burgdorferi* sensu lato causing Lyme disease and *Rickettsia* spp. were also detected in *L. agilis* mites [31, 33, 34].

Due to its high abundance, wide geographical distribution and permanent parasitic lifestyle, *L. agilis* mite offers an opportunity to relate its population genetic structure to the spectrum of its hosts, as well as to the genetic structure of its co-distributed louse parasites [14, 17, 18], which are of a similar lifestyle (permanent ectoparasites), but different evolutionary origin (insects). The present study provides the first insight into the phylogenetic and population genetic relationships of *L. agilis* across its geographical range in the Western Palearctic based on data obtained from the cytochrome c oxidase subunit I (COI) marker. We aimed to (a) delineate the evolutionary lineages of *L. agilis* across its distributional range in the Western Palearctic and (b) elucidate the phylogenetic relationships of *L. agilis* congeners with other members of the *Laelaps* genus. Then we aimed to (c) clarify the degree of host specificity of *L. agilis* concerning its abundant hosts in Europe (*A. sylvaticus, A. flavicollis,* and *Clethrionomys glareolus*) and regarding a possible co-occurrence with other related taxa, such as the *L. clethrionomydis*.

## Results

### Sequence analysis

**Fig. 1:**
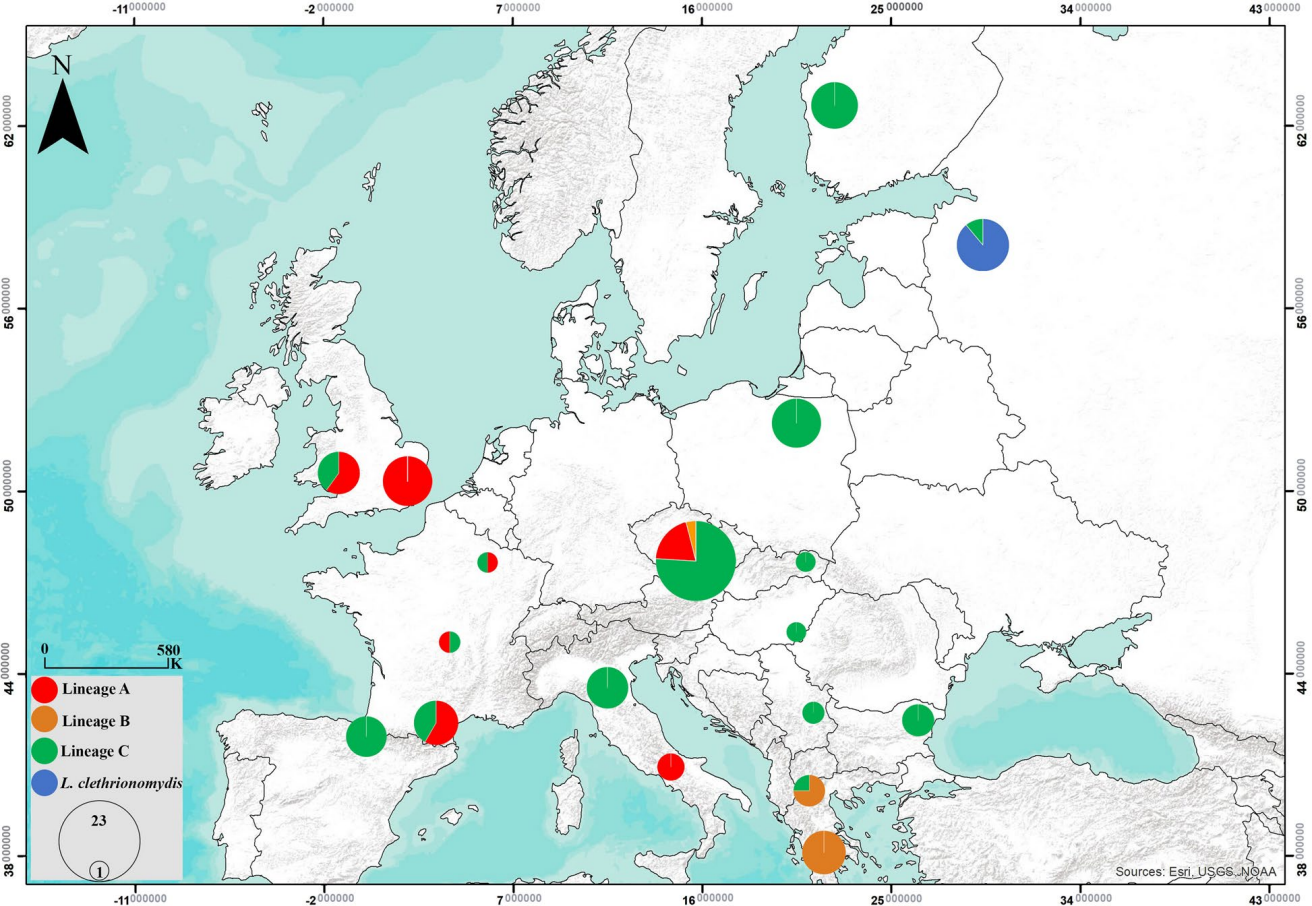
Map of sampled populations of *L. agilis*. Pie charts reflect the relative frequency of four mitochondrial lineages identified in studied populations. The size of the charts reflects sample sizes from one to 23 sequenced individuals per population

We separately analyzed two datasets containing COI sequences from *Laelaps* mites collected from four rodent host species across Europe (Fig. 1). The first dataset contained sequences of a short 381 bp fragment (Additional file 1). The short dataset was primarily used for population genetic analyses, whereas the second dataset contained 50 sequences of a longer 1026 bp fragment intended primarily for phylogenetic inference (Additional file 1). The 381 bp fragment of dataset 1 is included within the longer 1026 bp sequence of dataset 2.

The 132 sequences of the shorter dataset (381 bp) comprised 46 haplotypes. Sixty-seven sites (16.26% of total sites) were polymorphic, 57 of which were parsimony informative. The longer (1026 bp) dataset containing 50 sequences, formed 36 haplotypes, with 160 (15.09%) polymorphic sites, 146 of which were parsimony informative. Table 1 displays the genetic features of the populations comprising greater than or equal to three sampled individuals.

**Table 1:** Genetic statistics of *L. agilis* populations sampled in the Western Palearctic, based on the 381 bp COI fragment. Number of individuals (N), number of haplotypes (h), haplotype diversity (Hd), standard deviation (SD), nucleotide diversity (Pi), the total number of mutations (K), parsimony sites (P), and singleton sites (S)

Localities	N	h	Hd (SD)	Pi	K	P	S
Italy	13	8	0.923(0.05)	0.01831	24	21	3
France	35	8	0.803(0.041)	0.02274	23	18	5
Greece	7	5	0.905(0.103)	0.01925	15	14	1
Czech Republic	26	11	0.714(0.097)	0.02168	28	23	5
Austria	6	4	0.867(0.129)	0.00560	5	2	3
Germany	4	1	0 (0)	0	0	0	0
Bulgaria	4	3	0.833 (0.222)	0.00612	4	2	2
Finland	4	1	0 (0)	0	0	0	0
Spain	4	2	0.500(0.265)	0.00394	3	0	3
Poland	5	3	0.700 (0.218)	0.00315	3	0	3
United Kingdom	8	5	0.857 (0.108)	0.03075	25	22	1
Russia	3	2	0.66(0.314)	0.0035	2	0	2
Serbia^a^	2	-	-	-	-	-	-
Slovakia^a^	1	-	-	-	-	-	-
Hungary^a^	1	-	-	-	-	-	-

^a^Localities represented by a low number of samples, Slovakia and Hungary (one sample for each), and Serbia (two samples), were not included in the statistics

using the 381 bp dataset. Minimum and maximum nucleotide diversities were 0.000 (Finland and Germany) and 0.03075 (United Kingdom). Haplotype diversity ranged between 0.0 (Finland and Germany) and 0.923 (Italy). Moreover, the Czech Republic had the highest number of parsimony informative sites and singleton sites (Table 1). Neither stop codons nor insertions/deletions were observed in the datasets.

#### Phylogenetic analyses

Prior to phylogenetic reconstruction, we performed a saturation analysis, which showed that the long fragment of COI was a suitable marker for the analysis. The values of the substitution saturation index were smaller than the critical index of substitution saturation, indicating that our dataset has not experienced substitution saturation (Fig. S1 in the Additional file 2).

**Fig. 2:**
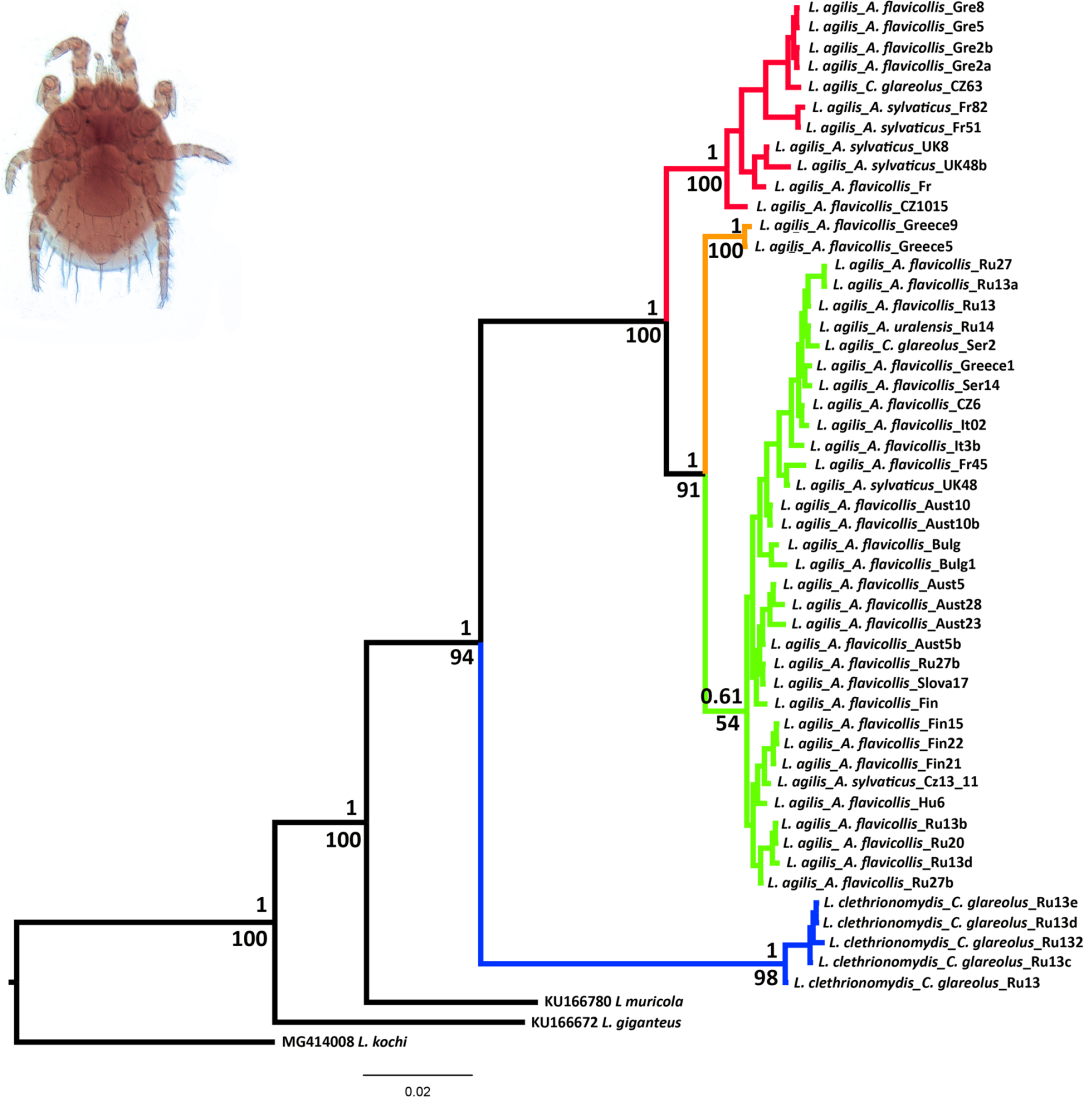
Phylogenetic tree reconstructed for *L. agilis* and *L. clethrionomydis* based on the 1026 bp fragment of COI gene. For each node, nodal supports indicate BI posterior probabilities (top) and ML bootstrap support (in percent, base). The image on the upper left shows *L. agilis*

Both methods employed in phylogenetic reconstruction (ML and BI) recovered identical topologies for evolutionary lineages of the two *Laelaps* species *(L. agilis* and *L. clethrionomydis*) sampled in the Western Palearctic (Fig. 2). Relative to the outgroups, *L. clethrionomydis* and *L. agilis* specimens clustered as sister monophyletic lineages with high posterior probability (1) and bootstrap support (94%). On the intraspecific level, *L. agilis* specimens formed three main lineages that diverged from each other with high support values. The first lineage (A) consisted of populations from Germany, the United Kingdom, France, Italy, and the Czech Republic. The second lineage (B) included two Greek specimens, and the third lineage (C) was geographically the most widespread, comprising populations from France, Italy, Czech Republic, United Kingdom, Slovakia, Serbia, Russia, Austria, Bulgaria, and Finland.

#### Molecular dating

Dataset 2 containing 50 novel sequences from Europe (1026 bp) was aligned together with 154 previously published haplotype sequences obtained from GenBank (644 bp) (Additional file 1). The sequences downloaded from GenBank belong to *L. kochi* (three sequences), *L. muricola* (55 sequences), *L. giganteus* (90 sequences), and *Dendrolaelaps* sp. (6 sequences) (Additional file 1).

**Fig. 3:**
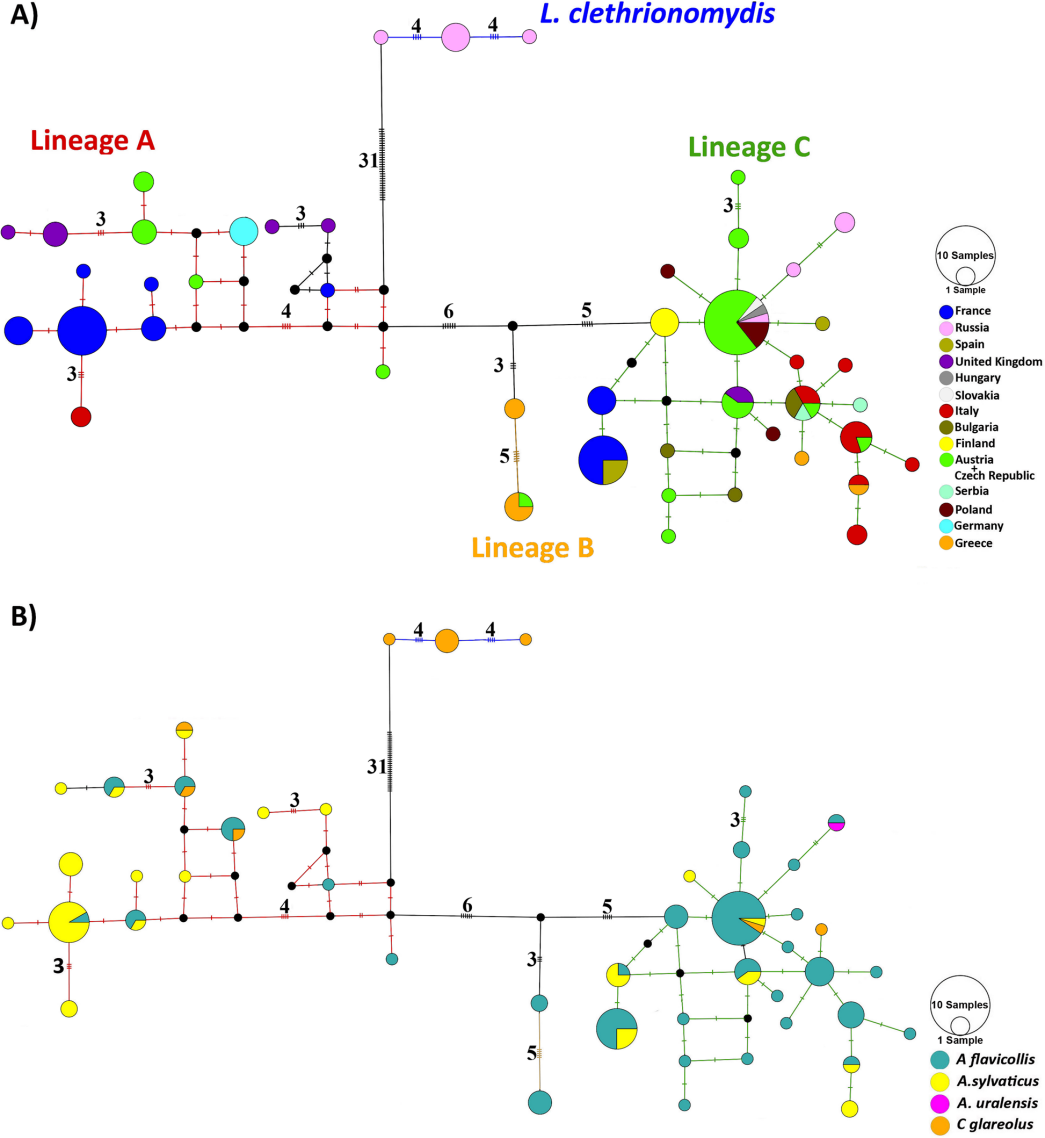
Chronogram resulting from dating analyses using the 1026 bp fragment of COI from 50 European *Laelaps* and sequences from GenBank (644 bp overlap, 166 sequences from African *Laelaps* species [19] and 6 outgroup sequences from *Dendrolaelaps* species comprising MG409996_*D. reticulosus*, MH983684_*D. presepum*, MH983831_*D. presepum*, MH983733_*D. presepum*, MH983835_*D. longiusculus*, MH983802_*D. longiusculus*). Chronogram was generated in BEAST v1.8.2. Branch numbers display times of divergence (Mya). The calibration point is indicated by a red circle, and the posterior probabilities of nodes are demonstrated by black squares and triangles

Divergence analyses estimated the origin of the genus *Laelaps* at approximately 11.96 Mya during the mid-Miocene (95% HPD: 11–12.94 Mya; Fig. 3). *L. kochi* emerged as the basal clade to the other four *Laelaps* species. The main lineages of *L. muricola* and *L. giganteus* diverged from *L. agilis* and *L. clethrionomydis* following subsequent cladogenic events dated to mid-Miocene at ∼8.86 Mya (95% HPD: 7.45–11.5 Mya; Fig. 3). The Lineage 1 *L. giganteus* discovered by previous studies [19, 21] separated from its Lineage 2 and *L. muricola* at ∼ 6.9 Mya, between late Miocene and early Pliocene (95% HPD: 4.71–7.81 Mya). The later cladogenesis between Lineage 2 *L. giganteus* and *L. muricola* was estimated to have happened during the Pliocene period at ∼ 4.72 Mya (95% HPD: 3.52–5.91 Mya). The divergence between *L. agilis* and its sister taxon, *L. clethrionomydis,* took place between the late Miocene and early Pliocene (5.87 Mya, 95% HPD: 3.63–7.56 Mya). Our molecular dating revealed that Lineage A split from the other two lineages of *L. agilis* during mid-Pleistocene at ∼ 1.79 Mya (95% HPD: 0.96–2.6 Mya). Finally, the last divergence within *L. agilis* occurred between Lineage C and Lineage B at 1.02 Mya (95% HPD: 0.58–1.82 Mya) during the late Pleistocene.

#### Spatial and non-spatial population genetic structure

**Fig. 4:**
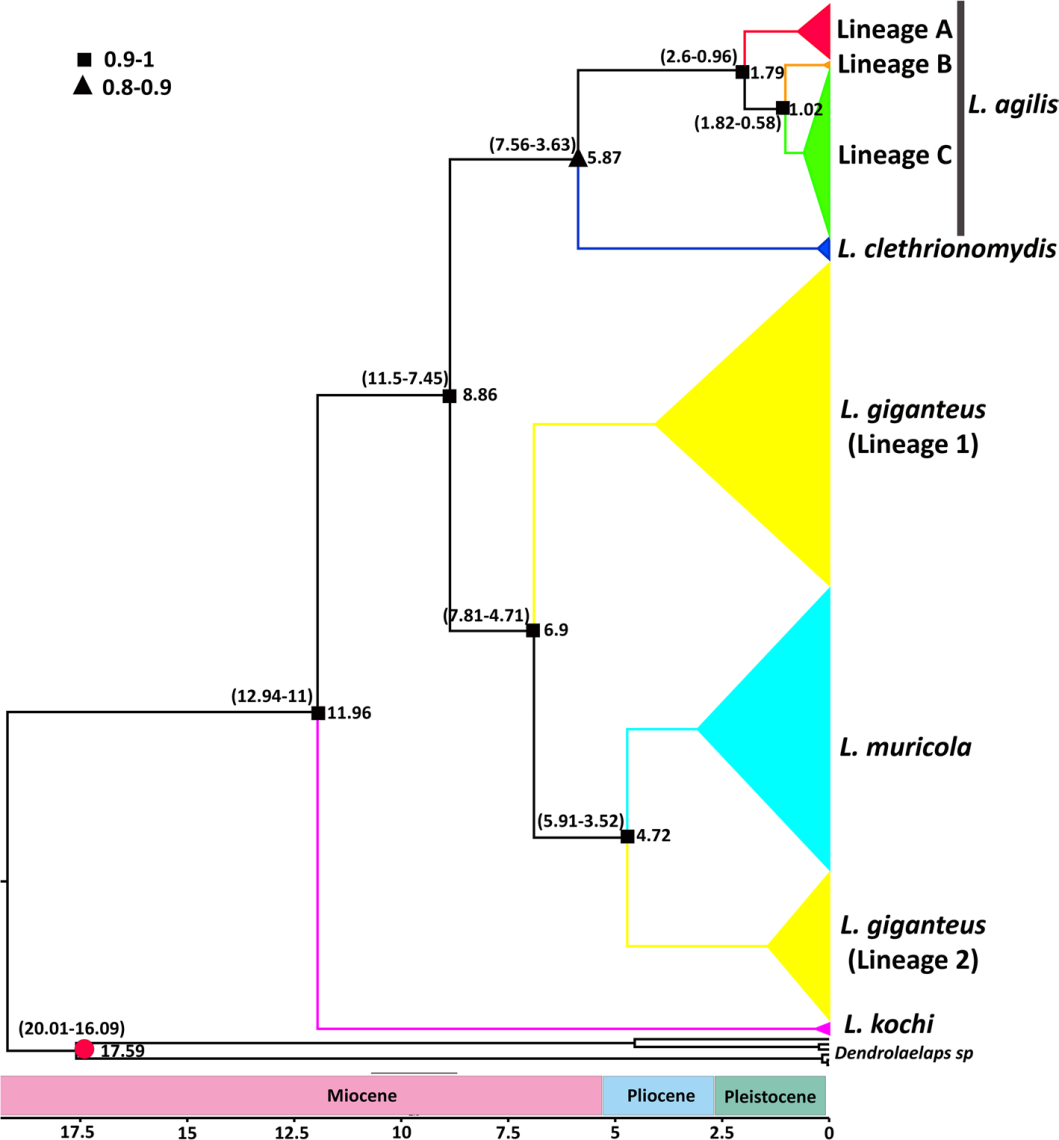
Haplotype networks of 126 *L. agilis* specimens from the Western Palearctic and six *L. clethrionomydis* representatives from Russia reconstructed using a 381 bp fragment of COI. The parasite populations were categorized based on their geographic areas (**A**) and their hosts (**B**). Dash symbols and numbers next to each line indicate the number of mutational steps. The size of the circles is proportional to haplotype frequencies. Putative unsampled haplotypes are shown by black circles. Geographical distribution of the lineages is provided in Fig. 1

Haplotype networks demonstrated the genealogy among *L. agilis* and *L. clethrionomydis* populations in the Western Palearctic (Fig. 4, Fig. S2 in the Additional file 2). The haplotype network based on the short fragment of COI gene recovered the same three haplogroups (lineages) as in the phylogenetic analysis of longer dataset 2, but with more specimens analysed. Haplogroup A included populations from western and central Europe (the United Kingdom, France, Italy, Germany, and the Czech Republic). The haplogroup A showed haplotype sharing between different hosts, but in contrast, no shared haplotypes were found among the populations from different geographic localities (Fig. 4). Haplogroup B comprised specimens from Greece and also one sequence from the Czech Republic. The haplogroup B was connected to haplogroup A with 8 and 17 mutational steps in the short and long fragment datasets, respectively (Fig. 4A and Fig. S2 in the Additional file 2). This haplogroup only included parasites from *A. flavicollis*, but its sample size was low (Fig. 4B). Haplogroup C split from the remaining lineages by 8 and 11 mutations in the short fragment dataset (381 bp, Fig. 4), and by 18 and 29 mutational steps in the long fragment dataset (1026 bp, Fig. S2 in the Additional file 2). This haplogroup not only included common haplotypes shared between different populations (Italy, France, Spain, Slovakia, Hungary, the Czech Republic, Poland, Bulgaria, Finland, Serbia, Greece, and the United Kingdom) but also comprised a common haplotype shared by the parasites from different hosts (*A. sylvaticus, A. flavicollis and C. glareolus*) (Fig. 4A and B). In both haplotype network analyses, the longest branch connected the *L. clethrionomydis* samples to *L. agilis* haplogroups (Fig. 4, Fig. S2 in the Additional file 2). *L. clethrionomydis* included populations only from Russia and *C. glareolus*, as in the phylogenetic analysis*.*

**Fig. 5:**
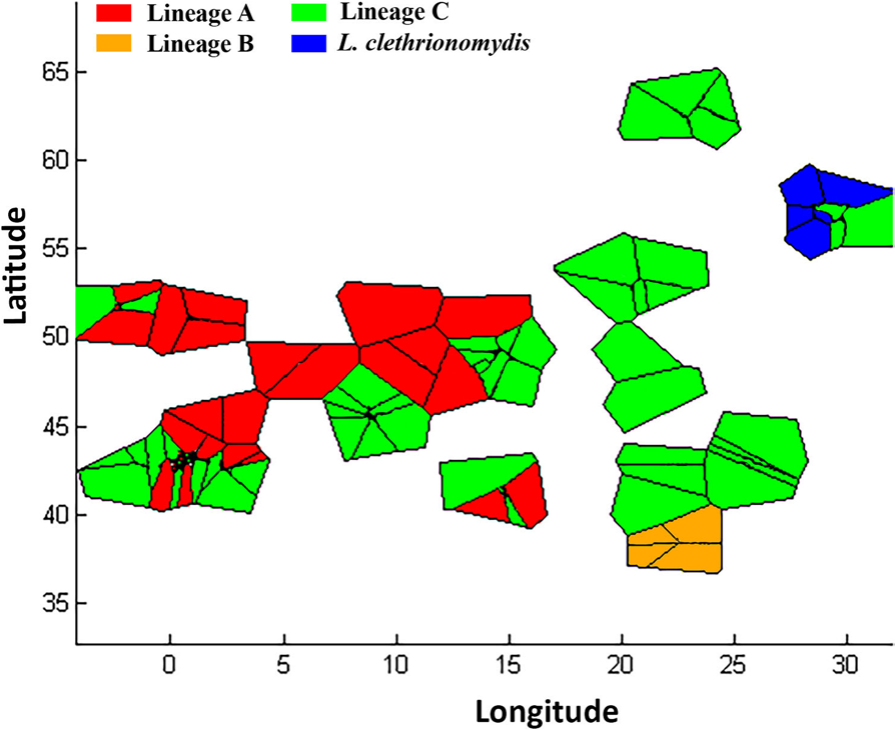
Bayesian spatial clustering of *Laelaps* lineages in the Western Palearctic conducted in BAPS for *K* = 4 clusters

The spatial population genetic structure of *Laelaps* in the Western Palearctic based on the short fragment dataset visualised by the BAPS is shown in Fig. 5. BAPS clustered all 132 samples into four groups consistent with the phylogenetic and haplotype network analyses: (i) cluster A (red trapezoids), including *L. agilis* populations from western Europe and the Czech Republic, (ii) cluster B (orange trapezoids), comprising populations from Greece and the Czech Republic, (iii) cluster C (green trapezoids), containing populations from Northern Europe (Finland), Eastern Europe (Serbia, Bulgaria, Slovakia, Hungary, the Czech Republic, and Poland), and Western Europe (Austria, Spain, Italy, France and the United Kingdom), and (iv) cluster D, including *L. clethrionomydis* samples (blue trapezoid, Fig. 5). Pairwise Fst proved significant genetic differentiation (*P* < 0.005) among the clusters, with values ranging from 0.67 (Lineage A and B) to 0.93 (Lineage A and *L. clethrionomydis*), Correspondingly, the highest and the lowest genetic distances were estimated at 3.4% and 13.3% between the same pairs of lineages as for the Fst (Table 2). The result of AMOVA analysis demonstrated that the majority of molecular variation in Lineage A was assigned among populations (54.91%) compared to 46.70% for Lineage C; On the contrary, the majority of genetic variation in Lineage C was explained within populations (54.30%) compared to 40.48% in Lineage A (Table S2 in the Additional file 2).

**Table 2:** Pairwise values of Fst (below diagonal) and genetic distance (above diagonal) for the four main clusters identified by population clustering in BAPS. All pairwise comparisons were significant at *P* < 0.005

	*L. agilis*				*L. clethrionomydis*
	N	Lineage A	Lineage B	Lineage C	
Lineage A	79		3.4%	5.1%	12.8%
Lineage B	6	0.67		3.9%	12.2%
Lineage C	41	0.79	0.67		13.3%
*L. clethrionomydis*	6	0.93	0.89	0.88	

#### Demographic history analyses and neutrality test

**Fig. 6:**
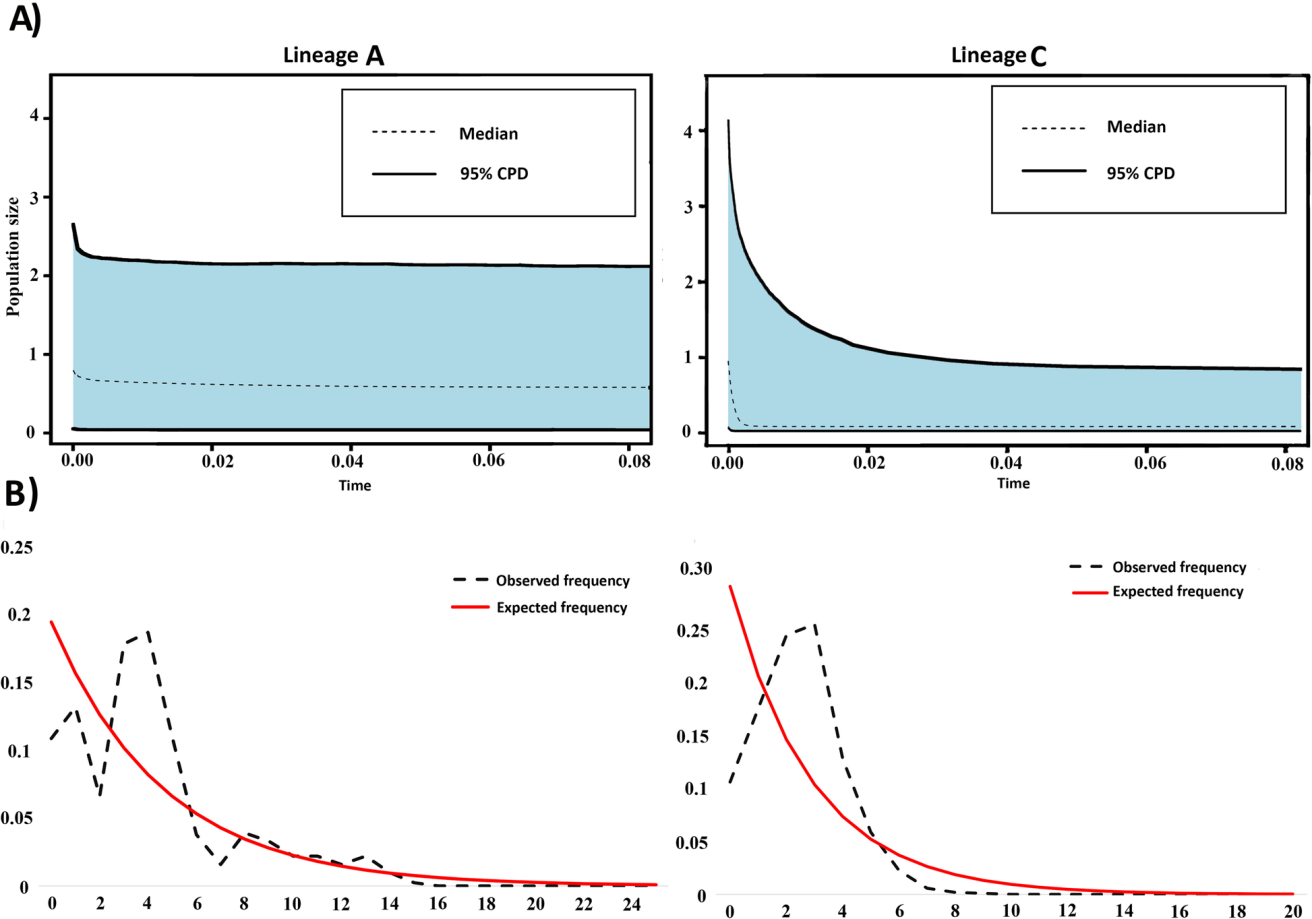
Demographic analyses. **A** Extended Bayesian Skyline Plot. The x-axis represents the time before present (Mya), while the y-axis demonstrates effective population size (Ne) per generation time. The dashed line depicts median values for the log10 of Ne. The grey shaded area expresses confidence intervals (95% HPD limits), **B** Demographic history of *L. agilis* is shown by mismatch distribution. Red line represents distributions under a constant population assumption. The black dotted line represents observed distributions

Results of the EBSP and mismatch distribution analyses for Lineages A and C based on 381 bp COI sequences were plotted in Fig. 6. We did not include Lineage B and *L. clethrionomydis* samples in demographic history analyses due to their low sample sizes. The results for Lineage A showed a constant population from 0.08 Mya up to the present day, and the mismatch analysis depicted a multimodal shape for this lineage (Fig. 6). In contrast, the EBSP plot for Lineage C showed a stable population size from 0.01 Mya to 0.08 Mya and a substantial increase in population size from 5000 years ago up to the present. Furthermore, the demographic analyses based on pairwise mismatch distribution revealed a unimodal mismatch distribution for Lineage C (Fig. 6b). In addition, Fu's Fs values were either negative (but not significant), based on the short fragment dataset, or positive based on the longer fragment for Lineage A. For Lineage C, significantly negative values of Fu's Fs were obtained analysing both the longer and shorter fragments of COI (Fu's Fs = -11.140 and − 16.55, respectively) (Table 3). Tajima D values were nonsignificant for both Lineages A and C.

**Table 3:** Neutrality tests for Lineages A and C in *L. agilis* based on shorter and longer fragments of COI. In bold, values significant at the *P* < 0.05 level

	Shorter fragment of COI (381 bp)			Longer fragment of COI (1026 bp)		
Populations	N	Fu ‘s Fs (*P* value)	Tajima D (*P* value)	N	Fu ‘s Fs (*P* value)	Tajima D (*P* value)
Lineage A	41	-3.73 (0.08)	-0.37 (0.14)	11	0.46 (0.11)	0.65 (0.14)
Lineage C	79	-**16.55 (0.01)**	-1.08 (0.10)	32	-**11.14 (0.002)**	-1.04 (0.11)

## Discussion

Our study concentrated on the reconstruction of both intra and interspecific relationships of *Laelaps agilis* and *L. clethrionomydis* mites, their host species, and geographic origin. Using a wide sample distribution across the Western Palearctic, we show that *L. agilis* is a monophyletic unit consisting of three evolutionary lineages. These lineages generally lack high host specificity and are found across a range of hosts (Fig. 3A). In addition, a fourth phylogenetically more distant lineage was found in sympatry with *L. agilis*. This lineage comprised of *L. clethrionomydis* samples collected from *C. glareolus* in the eastern range. Whilst none of the three *L. agilis* lineages showed clear specificity towards any of the host species, we found a striking difference in the depth of the genetic structure, particularly between a more geographically restricted Lineage A and a widespread Lineage C. The Demographic history reconstruction revealed that the two lineages differed also in their historical population sizes during quaternary glaciations. Below we discuss possible explanations of the observed patterns with regards to the distribution and quaternary evolution of *Laelaps* hosts compared to previous studies on co-evolution in rodent ectoparasites.

### Interspecific relationships within Laelaps

Despite using COI sequences from GenBank, we only had access to five species out of the 34 morphologically identified species of the *Laelaps* genus. Nevertheless, we were able to show that *L. agilis* forms a clear monophyletic unit, which was sister to *L. clethrionomydis*. These two European taxa clustered together and formed a sister group to African *Laelaps* lineages (Fig. 3). Based on 28S rDNA sequence data, Dowling and OConnor [35] revealed a polytomy structure in the phylogenetic relationships of 16 *Laelaps* species. In line with a previous molecular study [21], our results corroborate the polyphyly of the *L. giganteus* group. Furthermore, our phylogeographic reconstructions recovered *L. kochi* as the basal clade relative to other mite species, a finding in contrast to the study by Dowling and OConnor [35]. In addition to *L. kochi*, which forms the basal lineage in our dated tree, two major groups of taxa could be distinguished: (a) *Laelaps* species from Africa including two main lineages of *L. giganteus* and *L. muricola,* and (b) *Laelaps* species from Europe comprising *L. agilis* and *L. clethrionomydis.*

Previous studies have identified *L. muricola* as a generalist parasite species infecting several rodent species in South Africa [20, 21]. Also, very little genetic differentiation was observed among haplotypes of an *L. muricola* lineage from three native host species [20]. As opposed to the generalist *L. muricola*, its more host-specific relative, *L. giganteus,* occurs on two species of the two genera *Rhabdomys* and *Lemniscomys*. The result phylogenetic relationship from the dated tree is consistent with the previous study [21], showing that *L. giganteus* is paraphyletic with respect to *L. muricola*. Two cryptic lineages within *L. giganteus* were indicated as *L. giganteus* lineage 1 and *L. giganteus* lineage 2, with the lineage 2 grouped as a sister clade to *L. muricola*. Moreover, our divergence time revealed that at least cladogenetic events forming the lineages of *L. giganteus* lineage 1 and *L. muricola* + *L. giganteus* lineage2 took place already during the late Miocene and mid-Pliocene (at 6.9 and 4.72 Mya, respectively) suggesting an independent species status of each of the three lineages.

Our divergence time estimates showed that ancestors of African and European *Laelaps* species split at ∼8.86 Mya. Coincidentally, *Apodemus* separated from African *Malacomys* at 10.2 Mya [37]. Moreover, it has been indicated that the subfamily Murinae emerged during the mid-Miocene in southern Asia and expanded to Europe and Africa, evolving rapidly and dominating throughout the Late Miocene (~ 11 Mya) [38]. Additionally, the earliest *Apodemus* was discovered in Southern and Central Europe in early Vallesian, during Miocene (9.0–11.6 Mya) [39, 40]. It is evident that additional genetic data are needed from Asia and from different *Laelaps* species before a firm conclusion could be reached on the historical dispersion routes of the *Laelaps* genus.

Our results indicated that *L. agilis* separated from *L. clethrionomydis* during the late Miocene. In contrast to *L. giganteus* and *L. muricola* as facultative parasites spending most of their life cycles in the nests of their hosts, *L. agilis* and *L. clethrionomydis* are strictly haematophagous parasites that live on body fur, with females carrying eggs into the protonymph stage and laying them directly on the fur [32]. In our sampling, possibly due to a lower representation of *C. glareolus* specimens among captured mice, we recovered *L. clethrionomydis* only in western Russia. However, the species has been reported from Europe [41], northern Asia, and the Korean Peninsula to Japan, and it is considered not strictly host-specific [23]. Consequently, further molecular studies are required to clearly examine the host specificity and genetic diversity of *L. clethrionomydis* across its range.

### Population genetic structure and demography of L. agilis lineages

The result of the analysis of population genetic structure in *L. agilis* was in accordance with the phylogenetic trees, suggesting three significantly divergent groups across Europe (Fig. 2, Fig. 5, Table 2). Similarly, previous intraspecific studies have also reported splitting of other laelapid species into several cryptic lineages. Engelbrecht et al. [19] showed that *L. giganteus* populations diverged into six lineages with at least 11 mutational steps based on 644 bp of COI sequences, and two cryptic lineages were discovered for *L. muricola* in Southern Africa. Furthermore, high levels of intraspecific diversification or cases of possible sympatric speciation were previously found in other ectoparasites sharing rodent hosts with *Laelaps*, particularly in *Polyplax serrata* lice from European *Apodemus* spp. [14] or in *P. arvicanthis* from African *Rhabdomys* spp. [42].

Our analyses of demographic history and population structure revealed considerable differences between the two more common *L. agilis* lineages (A and C). EBSP results indicated that Lineage A maintained a constant population size. The multimodal mismatch distribution analysis suggested a diminishing or structured population, and, lastly, none of the neutrality tests showed significant values. Therefore, the hypothesis of constant population size for Lineage A cannot be rejected. In contrast, Lineage C expanded very recently, between 1,000 and 5,000 years (Fig. 6) before present, in the Meghalayan Age, the latest stage of the Holocene epoch [43]. Also, the mismatch distribution suggested a unimodal distribution for Lineage C, signifying a panmictic population that has experienced a sudden demographic expansion [44, 45]. Moreover, the star-like topology of the haplotype network exhibits a sudden population expansion [44]. Finally, both Fu's Fs and Tajima's D yielded significantly negative values for Lineage C, suggesting that the lineage has undergone at least one expansion event in its evolutionary history (Table 3).

Several studies have revealed a strong correlation between parasite phylogenies and their host genealogies [19, 46]. Using an *Apodemus*/*Polyplax* model, Martinů et al. [14] demonstrated that two widely distributed sister lineages of *Polyplax serrata* louse (N and S) both infect *A. flavicollis*; however, lice of the S lineage were shown to be strictly host-specific, whereas lice of the N lineage were not limited to a single host and exploited another host species, *A. sylvaticus*. Moreover, Martinů et al. [14] suggested that lice of the S lineage cannot be found on *A. sylvaticus* owing to adaptive specializations rather than due to the absence of host-switching opportunities. Whilst the high level of host specificity in lice could be associated with their limited capability of active dispersal between host species that do not occur in close contact, *Laelaps* mites, despite their host-bound lifecycle and bloodsucking diet, represent a more mobile type of parasite. During our sampling surveys, mites often left the host and actively searched the area around it, whereas the lice were incapable of any movement when off the host (personal observation). In accordance with these facts, the present study on *Apodemus*/*Laelaps* recovered two non-specific lineages (A and C) for *L. agilis* that infect *A. sylvaticus, A. flavicollis,* and the unrelated *C. glareolus* species. Although Lineage B of *L.agilis* was restricted only to *A. flavicollis,* it was represented by a small number of samples in our study, and a more comprehensive sampling is required to confirm or disprove its narrow host specificity.

Relating host specificity with the genetic diversity of populations, Li et al. [47] introduced the concept of the specialist-generalist variation hypothesis (SGVH). The hypothesis suggested that, in terrestrial systems, specialist parasite species show more subdivided population structures due to the patchiness of their habitats and host availability, leading to lower levels of genetic variation. Generalists, on the other hand, show lower levels of population structure [20, 48, 49] and higher genetic diversity compared to their specialist counterparts. Matthee et al. [20] demonstrated that the generalist parasite species *L. muricola* showed moderate levels of population differentiation (mtDNA Fst = 0.56, *p* < 0.05) and high mtDNA haplotype diversity of 0.97 (± 0.00), whereas the specialist species *L. giganteus* showed higher levels of population differentiation (COI Fst = 0.87, *P* < 0.05) and lower haplotype diversity of 0.77 (± 0.03). Based on such criteria, both A and C lineages of *L. agilis* could be regarded as generalists as they were characterized by high haplotypic diversities (Lineage A = 0.891 ± 0.035; Lineage C = 0.894 ± 0.023) and low to moderate levels of genetic differentiation (Fst, Lineage A = 0.56, *P* < 0.05; Lineage C = 0.46, *P* < 0.05). However, as we showed here, generalist or specialist life strategies, or the differences in dispersal capabilities (low in lice, higher in mites) are not the sole drivers of diversity in parasite populations. *L. agilis* Lineage A exhibited a more geographically subdivided population structure with no shared haplotypes among its geographic populations, in contrast to the highly admixed Lineage C (Fig. 3A, Table S2 in the Additional file 2). This finding highlights that other (historical) factors strongly contribute to genetic diversity in populations of parasites that otherwise show biologically similar features (same host spectrum and lack of visible morphological adaptations).

Our dated tree revealed that radiation between 1.79 and 1.02 Mya during the Pleistocene led to the formation of the three main lineages found in *L. agilis*. This finding supports Avise's [50] and Hewitt's [51] hypotheses that the intraspecific differentiation in many European species primarily emerged during the Quaternary period (Pleistocene and Holocene). Similarly, Nieberding et al. [52] demonstrated that the intraspecific structure of *Heligmosomoides polygyrus* associated with *A. sylvaticus* developed between 2.5 and 1.5 Mya in the Pleistocene period. Our *L. agilis* sampling found no host-specific lineages (exception for the rare Lineage B), suggesting that the individual lineages either maintained multi-host populations throughout their quaternary evolution or experienced multiple host switches following the glaciation period.

It has been validated that climate change gradually affected rodent communities between the Pleistocene and the Holocene [53]. The three common host species for *L. agilis* (*A. flavicollis*, *A. sylvaticus*, and *C. glareolus*) responded to and survived the Quaternary glacial periods in considerably different ways [54–57]. The occurrence of rodents across Europe thus varied during different periods. For most of the Late Pleistocene in western Europe, forest specialist species such as *C. glareolus* and *A. flavicollis* were not present but began to expand after the Last Glacial Maximum (LGM) [53, 58]. In contrast, forest-shrub generalists like *A. sylvaticus* were present during the Late Pleistocene and their habitats were preserved in places uncovered by ice [53, 58, 59].

Intraspecific genetic diversity of European *Apodemus* species shows differences attributable to their evolution in separated refugia. *A. sylvaticus* formed six mitochondrial lineages (African, Channel Islands, central, peripheral, south-eastern, and Sicilian lineages), separated by low levels of genetic distance and with overlaps in their central European range [56]. According to the divergence time analysis mtDNA lineages of *A. sylvaticus* diverged from each other between the late Pleistocene and early Holocene. Similarly, the populations of *A. flavicollis* comprise three major lineages separated by low levels of genetic divergence and little overlap in their distributions [55]. In addition, multiple glacial refugia, including a rather northern Carpathian refugium, have been described from different parts of *C. glareolus’* geographical distribution for eight divergent mtDNA clades [54]. The rapid range expansion of Lineage C in *L. agilis* was likely affected by the historical distribution of three host lineages, including Lineage 1 in *A. flavicollis* (see [57]) and peripheral lineages in *A. sylvaticus* [58]. In contrast, the genetic structure of Lineage A in *L. agilis* appears to be influenced by the central lineage in *A. sylvaticus* [56]. Glacial refugia in the eastern Pyrenees are a possible origin of Lineage A in *L. agilis*, while the Carpathians might be the source of the Lineage C. To be thoroughly tested, each of these hypotheses on historical co-evolution requires further sampling, with an emphasis on glacial refugia.

## Conclusions

Our analyses revealed unexpected genetic differences between two cryptic lineages of *L. agilis*. Whilst current populations of the Lineages A and C lack host specificity, the origin of these lineages and their genetic characteristics could be attributed to their historical co-evolution with one or several host species during Quaternary glaciations. Even though our current data do not allow for a detailed analysis of the co-evolutionary patterns associated with individual refugia, our results demonstrated how differently these historical processes may impact otherwise closely related evolutionary lineages of a single parasite species. Finally, by revealing the lack of host specificity in *L. agilis* lineages, our study provided new insight into the evolution of ectoparasitic lifestyle. Whereas *Polyplax serrata* louse species, sharing the same hosts but possessing lower capability for active dispersal, developed a much stricter level of host specificity (with one to two hosts per each of its mtDNA lineages), no such host-specific lineages were formed in the more mobile *L. agilis*.

## Material and methods

### Samples collection

Mice were captured using wooden snap traps in the frame of our previous research [14, 17, 18]. Host samples (fingertip or ear tissue) were collected and stored in ethanol, and the animals were visually checked for ectoparasites and combed. Mites were preserved in absolute ethanol at freezing temperatures. In total, 132 *Laelaps* specimens (126 samples of *L. agilis* and 6 samples of *L. clethrionomydis*) from four host species (*A*. *sylvaticus, A. flavicollis, A. uralensis* and *C. glareolus*) were collected from 12 European countries between 2005 and 2020 (Fig. 1, Table 1 and Additional file 1).

### DNA isolation, amplification, and PCR

Laelapid mites were individually isolated using QIAamp DNA Micro Kit (Qiagen) according to the protocol and DNA was eluted into 30 μl of AE buffer. After DNA extraction, mite exoskeletons were kept in 70% ethanol for subsequent use as vouchers. Partial sequences of the mitochondrial cytochrome oxidase subunit I gene (COI, 381 bp) were amplified for 132 specimens using primers L6625 and H7005 [60]. These primers were chosen to outline the picture of mite genealogy and population diversity. In order to clarify relationships among lineages of laelapid mites, we also sequenced a longer fragment of the COI gene (1026 bp) for a subset of samples (*n* = 50) using the COI primers LCO1490 and H7005 [61]. PCRs were carried out in a 25 ul volume using 1 ul of extracted DNA, 1 ul of each primer (at a concentration of 10 pM), 12.5ul 2 × concentrated PPP Master Mix (Top-Bio, CZ) and 9.5 ul of molecular grade H_2_O. The amplification protocol consisted of one denaturation step at 95 °C for 3 min, then 35 cycles of denaturation at 95 °C for 1 min, annealing at 50 °C (COI, 381 bp)/48 °C (COI, 1,026 bp) for 45 s and an extension step at 72 °C for 1.5 min, followed by the last elongation step at 72 °C for 10 min. PCR products were enzymatically cleaned up in a single-step process with VWR ExoCleanUp FAST PCR reagent (VWR, USA) following the manufacturer´s protocol. Purified PCR products were sequenced using the PCR primers in a commercial laboratory (Seqme, CZ). All sequences were deposited in GenBank (Additional file 1).

Due to overlapping morphometries of the two most abundant host species (namely *Apodemus flavicollis* and *A. sylvaticus*), it was often impossible to determine them unequivocally to the species in the field. All *Apodemus* samples used in this study were molecularly identified and obtained sequences published in the frame of our previous studies [14, 18, 62]. In short, host DNA was extracted from a host tissue sample using the DNeasy Blood & Tissue Kit (Qiagen, Hilden, Germany) according to the manufacturer’s instructions. A mitochondrial DNA control region sequence was obtained following PCR conditions as in Bellinvia [63]. PCR products were enzymatically purified and directly sequenced by Seqme (CZ).

### Sequence analysis

The Geneious Prime V2021.1.1 software was used to assemble and edit the sequences (https://www.geneious.com). ClustalW in MEGA V5 [64] was used to create two separate multiple sequence alignments for the short (381 bp) and longer (1026 bp) fragments. Then, sequences were translated to protein, based on invertebrate mitochondrial genetic code (translation table 5), to check for possible stop codons. Next, using DAMBE v6.0.4, substitution saturation of sequences was analyzed [65, 66]. DnaSP V5.0 [67] was used to assess nucleotide diversity, haplotype diversity, and sequence polymorphisms. Genetic distances were computed using the uncorrected pairwise genetic distances with 1,000 bootstraps in MEGA software V5. Also, nucleotide compositions, transition/transversion ratios and pairwise uncorrected distances were calculated in MEGA software V5.

### Phylogenetic analyses

The second dataset with longer fragments was used to reconstruct the phylogenetic relationship of *L. agilis* populations by Bayesian inference (BI) and Maximum Likelihood (ML) approaches. Using PartitionFinder V2.1.1 [68, 69], we identified the appropriate model (a) COI-codon 1, (b) COI -codon2, and (c) COI-codon3 for which the best fitting models were found to be HKY + G, SYM + G, and F81 + I, respectively, based on the Bayesian Information Criterion (BIC). Bayesian phylogenetic analyses were performed in MrBayes V3.2.2 [70] using the selected model of sequence evolution. We ran four parallel Monte Carlo Markov chains (MCMC) for 40 million generations, with trees being sampled every 1000 generations. Trace files were checked in Tracer V1.7 [71] and the first 25% of the generations were discarded as burn-in. Then, by combining the post-burn-in trees, a 50% majority-rule consensus tree was constructed. To estimate support of the Bayesian tree, we computed Bayesian posterior probabilities (PP). ModelFinder2 [72] was used to find the best-fitting model (GTR + F + I + G4) for IQTREE V2.1.2, which was then used to conduct maximum likelihood analyses and construct an ML tree [73]. Branch support of the ML tree was computed using 1000 ultrafast bootstrap replicates [74]. The -bnni option was applied to minimize the risk of overestimating support values. *L. kochi, L. muricola,* and *L. giganteus* (GenBank accession numbers: MG414008, KU166780, and KU166672, respectively) were used as outgroups.

### Divergence time

Divergence dates among five species of the genus *Laelaps* (*L. agilis*, *L. clethrionomydis*, *L. muricola*, *L. giganteus*, and *L. kochi*) were calculated using BEAST V1.8.2 [75], based on a fossil mite of the genus *Dendrolaelaps* dating back 16 million years (Mya) [76, 77]. Divergence estimation was modelled using a lognormal prior with 16 Mya as a zero offset and both lognormal standard deviation and lognormal mean were set to 1, resulting in a 95% confidence interval of 16.52–30.8 Mya. Due to only a single calibration point available for the analysis, we also applied the suggested mitochondrial substitution rates for mites (10^−6^substitutions/site/Myr, [78, 79]) to reduce the level of error at shallow nodes [80]. For the dating analyses, PartitionFinder V2.1.1 was utilized to search for the fittest partitioning schemes and models of evolution. Furthermore, a birth–death process was implemented as it better fits multispecies sequence datasets [81]. Uncorrelated lognormal was used as a clock model (an uncorrelated exponential clock also yielded similar results).

Three independent runs were conducted at 100 million generations, sampling every 5000 generations, and ultimately discarding the first 20% of the sampled trees as burn-in. Trace plots were visually inspected, showing good mixing of chains. Using Tracer V1.7, convergence was assessed by ensuring that effective sample sizes (ESS) above 500 were obtained. LogCombiner V1.8.2 was used to combine the parameter log and tree files. Then, TreeAnnotator V1.8.2 was utilized to generate a maximum clade credibility summary tree. To visualize and edit the resulting trees, FigTree V1.4.0 was used.

### Spatial and non-spatial population genetic structure

Population genetic relationships among *Laelaps* populations in the Western Palearctic were reconstructed via a median-joining algorithm in PopArt V1.7 [82], using short (381 bp) and long fragments (1026 bp) of COI separately. Using the short fragment dataset, a Bayesian-based method was performed in BAPS software V6.0 (Bayesian Analysis of Population Structure software) [83] to estimate the spatial clustering of individuals, and was followed by population mixture analysis. To detect the best value for the number of clusters (K) for population structure, we considered a range of 1–20 for values of K and the best-fit K was recognized through log marginal likelihood scores. Finally, Pairwise F-statistics were executed with 10,000 permutations in Arlequin V3.5 [84] for populations, which were grouped based on the best number of K. In addition, the stratification of genetic diversity (between individuals and populations) was estimated using the analysis of molecular variance (AMOVA) in Arlequin V3.5. The analysis was carried out separately for the two more common *L. agilis* lineages (A and C), which contained sufficient numbers of specimens and populations. Significance level of the statistics was obtained by 10,000 permutations of the data.

### Demographic history and neutrality test

Using BEAST V2.4.7, we created the Extended Bayesian Skyline Plot (EBSP; [85, 86]) in order to reconstruct the demographic history of *L. agilis* based on the short fragment dataset. We applied strict clock models using the same mutation rate exerted for the molecular clock. Following that, Bayesian MCMC chains were set at 900 million steps in total, with the Markov chain being sampled every 30,000 steps. Next, the convergence of MCMC runs, based on the effective sample sizes (> 250), was assessed using the Tracer program V1.5. Using the EBSP R script [87], the Bayesian skyline plots were generated in RStudio V4.0.1 [88].

Signatures of demographic or spatial population expansion in two major *L. agilis* populations were inferred using mismatch distributions in DnaSP V6.0. In addition, values of Tajima's D [89] and Fu's Fs statistics [90] were determined to assess demographic equilibrium in the Arlequin program V3.5. In these tests, negative values are caused by an excess of low-frequency polymorphisms, signifying size expansion and/or purifying selection [89].

## Supplementary Information

**Additional file 1.** 

**Additional file 2:** **Fig. S1.** Plot of the total numbers of transitions (s) andtransversions (v) against corrected distances based on GTR model revealed notrend toward saturation for transversions. All positions were employed in thesubsequent analysis. **Fig. S2.** Genealogic relationships (Median Joining network) of the *L.agilis* and L. *clethrionomydis,*based on 1026 bp of COI sequence. Numbers above the connecting branches reflectthe number of mutational steps joining the haplotypes (denoted by circles). Thesize of each circle is proportional to the number of individuals. **Table S2.** Analysis of molecular variance (AMOVA) forpopulations within lineages A and C.

## Data Availability

Sequence data allowing reconstruction of the results obtained in this study are available in the GenBank repository under accession numbers OM754564-OM754695.
