# Supplementary material for: Phylogeography of the parasitic mite Laelaps agilis in Western Palearctic shows lineages lacking host specificity but possessing different demographic histories
Source: BMC Zool. 2022 Mar 24;7:15. doi: 10.1186/s40850-022-00115-y (PMC10127304; doi:10.1186/s40850-022-00115-y)
Supplement: Supplementary file 2 — Additional file 2: Fig. S1. Plot of the total numbers of transitions (s) andtransversions (v) against corrected distances based on GTR model revealed notrend toward saturation for transversions. All positions were employed in thesubsequent analysis. Fig. S2. Genealogic relationships (Median Joining network) of the L.agilis and L. clethrionomydis,based on 1026 bp of COI sequence. Numbers above the connecting branches reflectthe number of mutational steps joining the haplotypes (denoted by circles). Thesize of each circle is proportional to the number of individuals. Table S2. Analysis of molecular variance (AMOVA) forpopulations within lineages A and C. [file 40850_2022_115_MOESM2_ESM.docx]

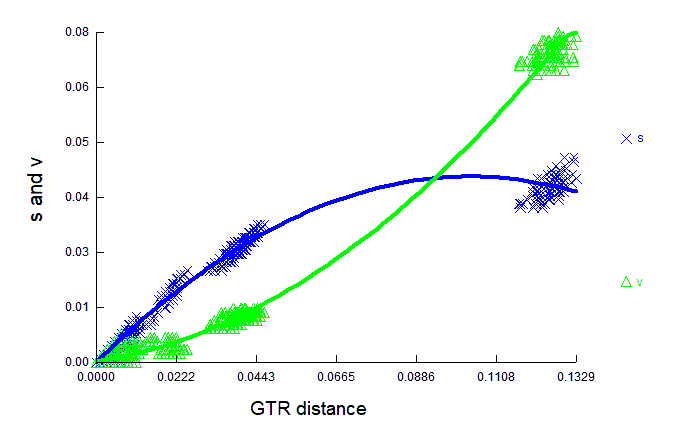


Fig. S1 Plot of the total numbers of transitions (s) and transversions (v) against corrected distances based on GTR model revealed no trend toward saturation for transversions. All positions were employed in the subsequent analysis.


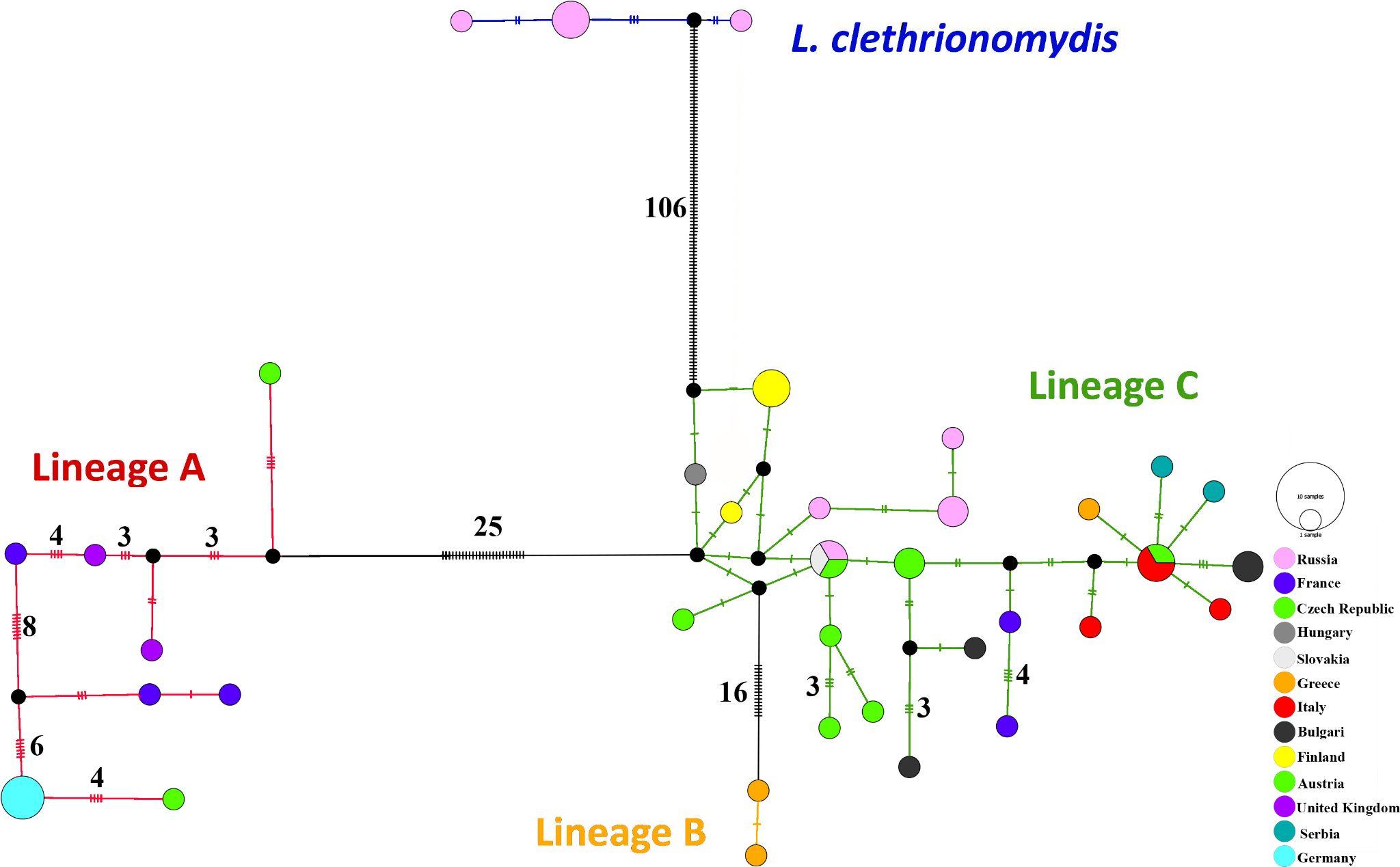


Fig. S2 Genealogic relationships (Median Joining network) of the *L. agilis* and L. *clethrionomydis,* based on 1026 bp of COI sequence. Numbers above the connecting branches reflect the number of mutational steps joining the haplotypes (denoted by circles). The size of each circle is proportional to the number of individuals.

Table S2 Analysis of molecular variance (AMOVA) for populations within lineages A and C

| **Statistics** | **Percentage of variation** | **Sum of squares** | **d.f.** | **Source of variation** |
| --- | --- | --- | --- | --- |
| FST = 0.55 | 54.91 | 42.48 | 3 | (**Lineage A**) Among populations |
|  | 45.09 | 40.48 | 36 | Within populations |
|  |  |  | 39 | Total |
|  |  |  |  |  |
| FST = 0.46 | 45.70 | 52.51 | 10 | (**Lineage C**) Among populations |
|  | 54.30 | 42.92 | 64 | Within populations |
|  |  |  | 74 | Total |
